# Microinvasion of liver metastases from colorectal cancer: predictive factors and application for determining clinical target volume

**DOI:** 10.1186/s13014-015-0428-2

**Published:** 2015-06-04

**Authors:** Yang Qian, Zhao-Chong Zeng, Yuan Ji, Yin-Ping Xiao

**Affiliations:** Department of Radiation Oncology, Zhongshan Hospital, Fudan University, 136 Yi Xue Yuan Road, Shanghai, 200032 China; Department of Pathology, Zhongshan Hospital, Fudan University, Shanghai, China

**Keywords:** Liver metastases, Colorectal cancer, Radiotherapy, Pathological characteristics, Tumor invasion

## Abstract

**Objectives:**

This study evaluates the microscopic characteristics of liver metastases from colorectal cancer (LMCRC) invasion and provides a reference for expansion from gross tumor volume (GTV) to clinical targeting volume (CTV).

**Methods:**

Data from 129 LMCRC patients treated by surgical resection at our hospital between January 2008 and September 2009 were collected for study. Tissue sections used for pathology and clinical data were reviewed. Patient information used for the study included gender, age, original tumor site, number of tumors, tumor size, levels of carcinoembryonic antigen (CEA) and carbohydrate antigen 199 (CA199), synchronous or metachronous liver metastases, and whether patients received chemotherapy. The distance of liver microinvasion from the tumor boundary was measured microscopically by two senior pathologists.

**Results:**

Of 129 patients evaluated, 81 (62.8 %) presented microinvasion distances from the tumor boundary ranging between 1.0 − 7.0 mm. A GTV-to-CTV expansion of 5, 6.7, or 7.0 mm was required to provide a 95, 99, or 100 % probability, respectively, of obtaining clear resection margins by microscopic observation. The extent of invasion was not related to gender, age, synchronous or metachronous liver metastases, tumor size, CA199 level, or chemotherapy. The extent of invasion was related to original tumor site, CEA level, and number of tumors. A scoring system was established based on the latter three positive predictors. Using this system, an invasion distance less than 3 mm was measured in 93.4 % of patients with a score of ≤1 point, but in only 85.7 % of patients with a score of ≤2 points.

**Conclusions:**

The extent of tumor invasion in our LMCRC patient cohort correlated with original tumor site, CEA level, and number of tumors. These positive predictors may potentially be used as a scoring system for determining GTV-to-CTV expansion.

## Summary

Data from 129 patients with liver metastases from colorectal cancer (LMCRC) were used to analyze factors associated with hepatic metastatic microextensions. The extent of invasion was positively correlated with the original primary tumor site, CEA levels, and number of tumors. These predictors may potentially be used as a scoring system for determining gross tumor volume (GTV) to clinical tumor volume (CTV) expansion.

## Introduction

Colorectal cancer is one of the most common gastrointestinal carcinomas, and worldwide incidence and mortality rates are continually increasing. The five-year survival rate after radical surgery is 40−56 %. The major failure pattern involves liver metastases. During follow-up observation, 50−70 % of patients diagnosed with colorectal cancer exhibit liver involvement, which presents as a solitary nodule in one-half of these patients [1]. In approximately 25 % of cases diagnosed with colorectal cancer, liver metastases are found at the time of diagnosis. Among those without liver metastases at the time of diagnosis, 80−90 % will have liver metastases within 2–3 years after surgery [2].

In 2010, colorectal cancer guidelines proposed that carefully selected patients should be considered for conformal radiation therapy and included in some clinical trials. Studies in the literature show that stereotactic body radiation therapy (SBRT) for liver metastases from colorectal cancer (LMCRC) is an effective ablation therapy with a favorable toxicity profile [3, 4]. Conformal radiotherapy accurately shapes the high-dose radiotherapy beam closely around the outline of the visible tumors and subclinical lesions. Precise outlining of the gross tumor volume (GTV) and the clinical targeting volume (CTV) can focus the radiation dose to the target tumor and reduce exposure and toxicity to normal tissue.

GTV is the volume of the visible tumor. CTV includes GTV and is defined as the volume of tissue that has a significant probability of containing microscopic tumor extensions (microextensions), which represents subclinical diseases. Few studies have analyzed microextension characteristics in liver metastasis. This knowledge deficit limits the application of conformal radiotherapy for LMCRC. The objectives of this study were to perform histological quantification of microextension at liver metastatic lesions, and to correlate pathological macroscopic tumor dimensions with MRI measurements to define CTV as precisely as possible.

## Methods

### Ethics approval and patients in the study

This study was approved by the ethical review board of Zhongshan Hospital, Fudan University and in compliance with the Helsinki Declaration of 1975, as revised in 2000. Between January 2008 and September 2009, 129 patients with liver metastasis from colorectal cancer underwent radical surgical resection at Zhongshan Hospital. Patient selection was based on the following criteria. Patients must have undergone a radical resection for liver lesions at our hospital, and must have been pathologically diagnosed with metastatic adenocarcinoma. To avoid potential influences on the presence of microextensions, patients may not have received recent local treatment for liver metastatic lesions. Complete clinical data must have been available for patients, including tumor information and laboratory values. Normal liver tissue surrounding tumors must have had at least a 1-cm margin extending beyond the tumor boundary, so that all microextensions could be observed properly. Many lesions are excluded from radiotherapy due to possible radiation damage of normal liver [5, 6]. Patients were excluded from the current study if more than four lesions were detected by preoperative radiography or laparotomy. If more than one lesion was present, we selected the largest one to evaluate.

### Pathological analysis

After radical resection, surgical specimens were analyzed by surgeons to determine the pathological boundary type and maximum diameter. Then, some specimens were fixed in phosphate-buffered saline (PBS) containing 10 % (v/v) formalin; in this study, these are designated as pathology specimens. Unfixed specimens are designated as surgical specimens. All specimens were sectioned into 1.5 × 1.0 × 0.4-cm slices, dehydrated, and then embedded in paraffin. Tissue blocks were prepared for routine histological examination, and 5-μm sections on slides were stained with hematoxylin and eosin (H&E) for light microscopy. Three hundred slides from these specimens were reviewed. To avoid variations between observations, a single pathologist assessed all specimens to identify microscopic evidence of microextension.

### Analysis and definitions of microextensions

**Fig. 1:**
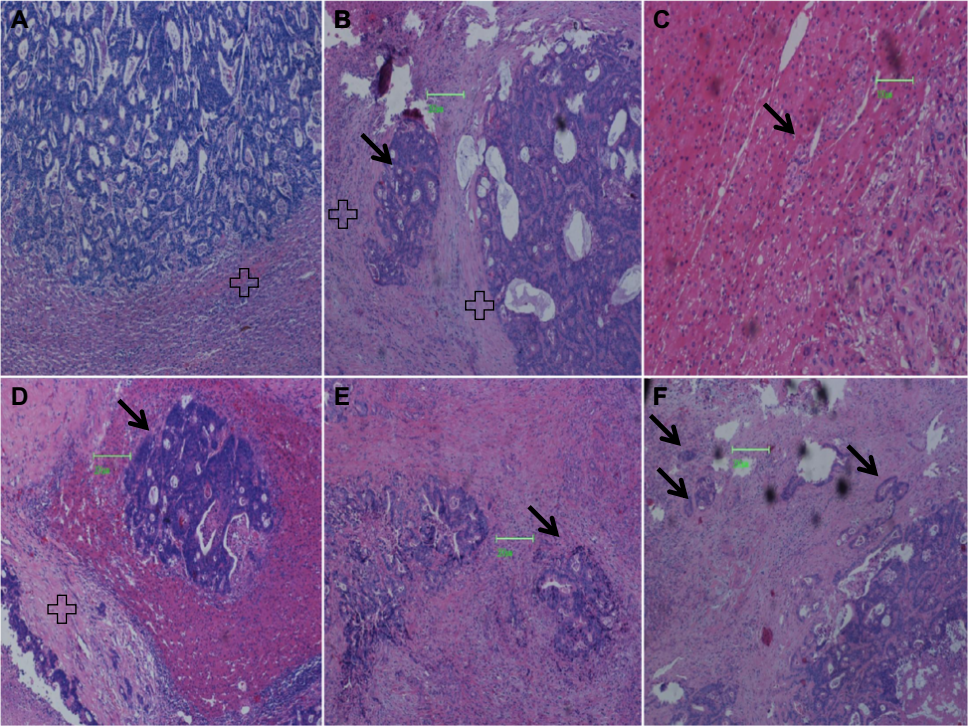
Survey of observations of liver microinvasion from colorectal tumor metastases. **a** Tumor with a capsule (black cross) and showing a distinct boundary. **b** Tumor and microinvasions (black arrows) with capsules. **c** Tumor and microinvasions without capsules. **d** Tumor with a capsule that does not exhibit microinvasions. **e** Irregularly protruding microinvasions. **f** Multiple microinvasions (black arrows) with different sizes and shapes that show obscure boundaries

On each histological slide, tumor margins were assessed on the cut surface by gross findings and then outlined by marker pen with indelible ink. A microextension was defined as an extension of the tumor through the marked margin, and was analyzed by light microscopy using an Olympus BX 40 (Olympus; Tokyo, Japan) at low-power magnification (×40) to identify the boundary between the tumor and normal liver tissues. These observations also were confirmed by examination at a higher magnification (×100 or × 400). On each slide, the maximum distance to normal liver tissue around the tumor periphery was determined using an eyepiece with micrometer. If two or more microinvasions were observed (Fig. 1f), the longest invasive distance was recorded. If the primary tumor was completely or partially surrounded by a fibrous pseudocapsule (Fig. 1b), the microextension was considered to be the maximum distance from the outer border of the pseudocapsule to the outer boundary of visible nests of tumor cells [7]. If a fibrous pseudocapsule surrounding the primary tumor was absent (Fig. 1c), the tumor mass was defined as the area that lacked (or essentially lacked) liver parenchyma interspersed between tumor cells as determined by microscopic examination. If the tumor had a capsule and did not exhibit microinvasion (Fig. 1d), the microextension was considered to be the maximum distance from the outer border of the pseudocapsule to the outer boundary of visible nests of tumor cells. The tumor border was defined as the line along whose length liver tissue and nests of tumor cells became interchanged; in this case, the microextension was defined as the distance from the primary tumor border to the outer boundary of visible nests of tumor cells (Fig. 1) [8].

### Magnetic resonance imaging and computed tomography

We collected data from magnetic resonance imaging (MRI) and computed tomography (CT) images to compare with the pathological measurements. To allow as little time as possible for additional tumor development before performing surgery, the scans had to be performed within 3 days before resection. Of the two imaging modalities, MRI was preferred to CT for its superior assessment of malignant focal liver lesions [9, 10].

**Fig. 2:**
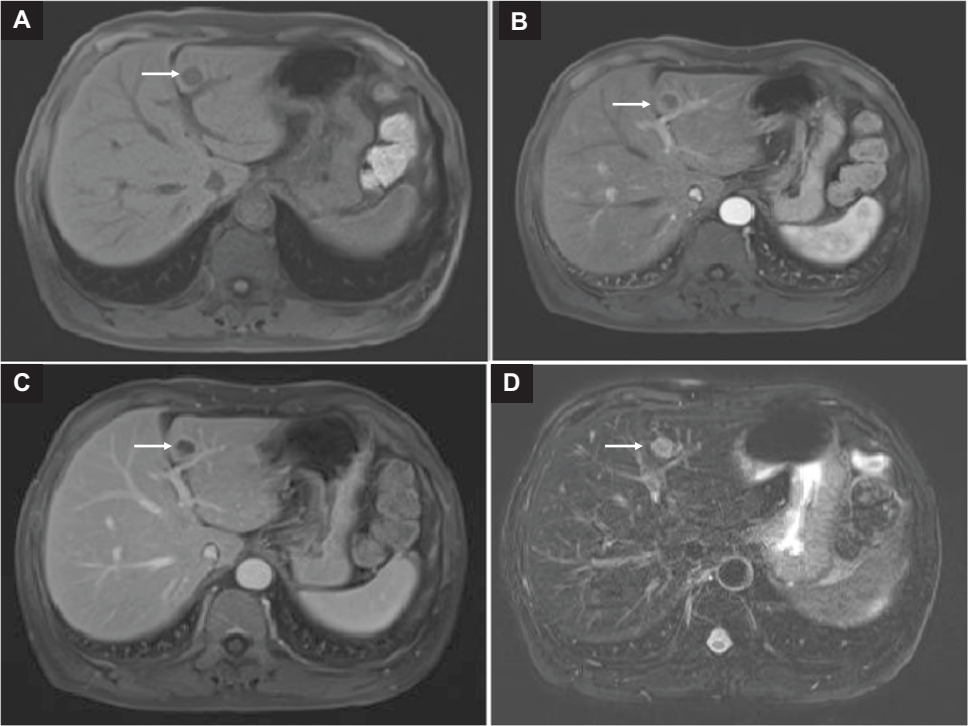
Liver metastasis (white arrow) of preoperative magnetic resonance images. **a** T1-w GE. **b** T1-w arterial enhanced. **c** T1-w venous enhanced. **d** T2-w

Of the 129 patients in this study, only 49 underwent MRI at our hospital. An MRI scan of the liver was performed with a 1.5 T MR scanner (SignaHDxt; General Electric, WI, USA). This preoperative MRI included a T2-weighted (w) fast-spin echo (FSE) single-shot (SS) sequence, a T1-w gradient-echo (GE) sequence, and a T1-w dynamic multiphase gadolinium-enhanced (DMGE) sequence (Fig. 2a–d). All MRI sequences were performed while the patient held his breath (after exhalation). The T2-w FSE FS sequence also was performed with the system triggered to expiration. The slice thickness for T1-w GE and T2-w FSE SS sequences was 8 mm, and for the T1-w DMGE sequence it was 5 mm. Liver metastases vary in their T1 and T2 signal intensities but are usually prolonged, which results in hypointensity to isointensity on T1-w images and isointensity to hyperintensity on T2-w images. Some metastases may show a hyperintense halo of viable tumor surrounding a central region of hypointensity due to necrosis. The pathologically determined GTV correlated best with unenhanced MRI compared with venous enhanced MRI. The tumor contours revealed by T1-w GE were reviewed by an experienced liver radiologist.

### Comparison of preoperative MRI with macroscopic pathology

To define the GTV by radiography, we calculated that the coefficient of specimen contraction during fixation for a pathology slide was 90.1 %, as reported by Bi *et al.* [11] from their analysis of 66 primary liver carcinoma specimens. The pathology specimens, which included both tumor and normal liver tissues, were cut into a regular rectangle of 2 × 1.5 cm. The width and length of the cut tissue were measured. Two to four pieces of the specimens were placed into an automatic tissue hydroextractor and subjected to a dehydration cycle. H&E-stained slides were prepared using routine methods, and the resulting tissue sections were measured. Finally, the specimen contraction coefficient was calculated based on the two measured specimen sizes.

### Definitions of clinical factors

The preoperative evaluation included a medical history, physical examination, complete blood-cell count, liver function tests, measurements of carcinoembryonic antigen (CEA) and carbohydrate antigen 19–9 (CA199) levels, chest X-ray, abdominal ultrasonography, and enhanced CT or MRI (or both). Levels of CEA and CA199 tumor markers were measured using an electrochemiluminescence immunoassay system (Roche Modular E170). Liver functions were tested by an automated method and analyzed statistically (Hitachi 7600–120). All laboratory tests were performed less than 1 week before surgery at the Clinical Laboratory Department of Zhongshan Hospital. The presence of multiple lesions is defined as more than one lesion but less than four lesions. Chemotherapy is defined as having undergone at least one chemotherapeutic regimen before live surgery. Liver metastases are defined as synchronous when they occur within 6 months after the original tumor surgery and as metachronous when they occur after 6 months. The surgeons specified the original tumor site as rectum when it was located within <12 cm from the perianal skin border, and as colon when it was located in cecum, ascending colon, transverse colon, descending colon, or sigmoid colon. All patients had hepatic lesions that were consistent with metastases from a histologically documented colorectal carcinoma.

### Statistical analysis of data

Statistical analysis was performed using SPSS version 19.0 (SPSS Inc., Chicago, IL). The association of characteristics with microinvasion distance was analyzed by chi-square test for categorical variables, and by binary logistic regression for predictor variables of the significant parameters. *P* values ≤0.05 were considered significant.

## Results

### Demographic data

One hundred and twenty-nine LMCRC patients who fulfilled the specified criteria were recruited, including 75 men and 54 women. The patient ages ranged from 32 to 80 years, with a mean of 59 years.

Microextension was noted in 62.8 % (81/129) of the patients. Microinvasion was assessed from H&E-stained sections. Microscopic analysis of tissue specimens from these patients revealed two microinvasion patterns in LMCRC patients. In one, the region of microextension and tumor foci was separated by normal liver tissue (Fig. 1b). The second pattern was irregular protrusion extending beyond the tumor (Fig. 1e). The microextension distances ranged from 1–7 mm, with an average distance of 1.50 ± 1.51 mm. The microextension distance was 1–2 mm in 48 (37.2 %) patients, 2.1 − 4.0 mm in 24 (18.6 %) patients, and 4.1 − 7.0 mm in 9 (7 %) patients. This distance had to be expanded to 5 mm during microscopy to include at least 98 % of all microextensions.

### Factors associated with the presence of microextensions

Clinical factors associated with the presence of microextension are listed in Table 1. The data indicated that the presence of microextensions was associated with high serum levels of CEA (*P* = 0.002), primary tumor site in colon (*P* = 0.008), and multiple lesions (*P* = 0.045).

**Table 1:** Factors associated with the presence of microextension

Clinicopathological factor	*n*	Microextension, *n* (%)		*x* ^2^	*P*-value*
		Absent	Present		
Sex				0.790	0.598
Male	75	28 (37.3 %)	47 (62.7 %)		
Female	54	20 (37.0 %)	34 (63.0 %)		
Age				0.794	0.624
<65 years	93	34 (36.6 %)	59 (63.4 %)		
≥65 years	36	14 (38.9 %)	22 (61.1 %)		
Synchronous or metachronous				0.982	0.965
Synchronous	70	25 (35.7 %)	45 (64.3 %)		
Metachronous	59	23 (39 %)	36 (61 %)		
Original site				3.185	0.009
Rectum	48	25 (52.1 %)	23 (47.9 %)		
Colon	81	23 (28.4 %)	58 (71.6 %)		
Number of tumors				2.537	0.045
1	85	38 (44.7 %)	47 (55.3 %)		
>1	44	10 (22.7 %)	34 (77.3 %)		
Max tumor diameter				1.345	0.52
<3 cm	39	17 (43.6 %)	22 (56.4 %)		
≥3 cm	90	31 (34.4 %)	59 (65.6 %)		
CEA (ng/ml)				4.805	0.001
≤5	35	20 (57.1 %)	15 (42.9 %)		
>5	94	28 (29.8 %)	66 (70.2 %)		
CA199 (U/ml)				0.478	0.114
≤37	68	24 (35.3 %)	44 (64.7 %)		
>37	61	24 (39.3 %)	37 (60.7 %)		
Chemotherapy				0.906	0.817
Yes	55	21 (38.2 %)	34 (61.8 %)		
No	74	27 (36.5 %)	47 (63.5 %)		

*CEA* Carcinoembryonic antigen, *CA199* Carbohydrate antigen 19–9

*Determined by binary logistic regression

### Scoring system to estimate risk of microextensions with varying distances for all patients

**Fig. 3:**
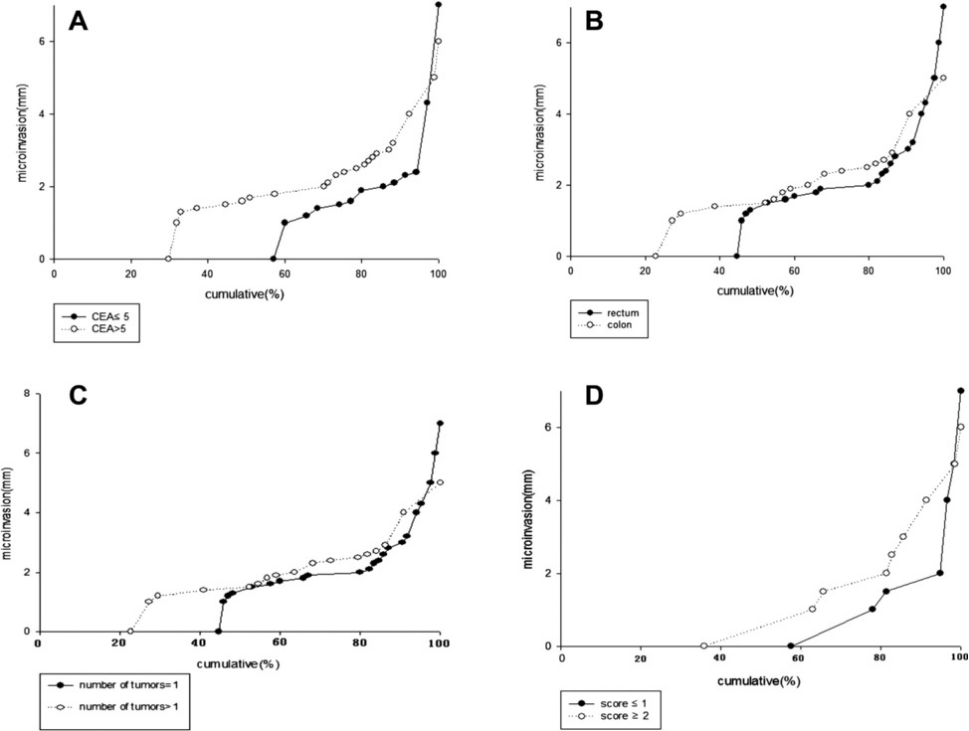
Cumulative distributions of LMCRC microextensions according to serum CEA level. **a** Serum CEA levels. **b** Original primary tumor site. **c** Intrahepatic tumor number. **d** A score derived from a combination of these factors

Plots showing the cumulative distribution of microinvasion according to the clinicopathologic factors are presented in Fig. 3. We found that among patients with microinvasion distance of ≤2.4 mm, 94.3 % had CEA levels of ≤5 U/ml. When the microinvasion distance was ≤4.3 mm, sensitivity was approximately 95 % for patients in which the primary tumor site was rectum. A microinvasion distance of ≤4 mm was associated with a sensitivity of 95 % for patients with a single liver metastatic lesion (unilesional). The best single predictor for microinvasion was the CEA level.

To improve the success of prediction for microinvasion, we developed a scoring system based on a combination of the associated factors that were identified. Patients were assigned a score of 0 for each of the following: CEA level ≤5; original site of primary tumor in rectum; and unilesional liver metastasis. Otherwise, patients received a score of 1 for factors not meeting those criteria. To account for ≥95 % probability of microinvasion, one must select an expansion of 2 mm for a score of ≤1, and an expansion of 5 mm for a score of ≥2 (Fig. 3d).

### Tumor sizes determined from radiographs, surgical specimens, and pathology specimens

**Fig. 4:**
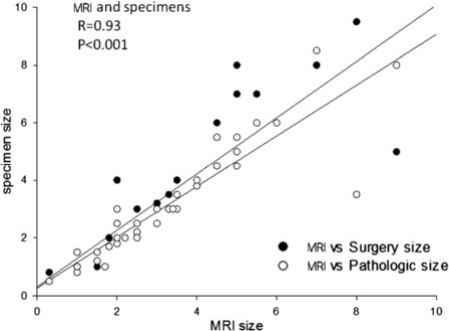
Correlation between tumor sizes measured using magnetic resonance images and pathology specimen slides

In samples from the 49 patients undergoing a preoperative MRI scan at our hospital, only 49/129 tumors (38 %) received the same boundary classifications based on gross analyses of surgical specimens and radiographs. The mean tumor diameter measured 3.3 cm (range, 0.3–9.0 cm) in radiographs, 3.5 cm (range, 0.8 − 9.5 cm) in surgical specimens, and 3.2 cm (range, 0.8–9.5 cm) in pathology specimens. Regression analysis of tumor size in radiographs, surgical specimens, and pathology specimens revealed that the three measurements were significantly correlated (*P* < 0.001). This was especially true of measurements taken from radiographs and surgical specimens. Measurements from pathology specimens were slightly smaller than those taken from radiographs (*r* = 0.92, *P* < 0.001). A comparison of radiographic measurements with those obtained from pathology specimens showed that radiographic measurements were larger in 34.7 % (17/49) of tumors, smaller in 40.8 % (20/49) of tumors, and identical in 24.5 % (12/49) of tumors. Overall, measurements taken from pathology specimens were smaller than those obtained from radiographs (*r* = 0.92, *P* < 0.001; see Fig. 4), with pathology specimen measurements reduced by 8 % relative to those from radiographs.

The radiograph-slide contraction coefficient was 82.9 % (0.92 × 0.90), and a distance of 5 mm was required to include 98 % of microextensions. By extrapolation, the equivalent distance on radiographic specimens had to be 6.1 mm (5.0/0.829). For a score of ≤1, an expansion distance of 2.41 mm (2.0/0.829) on radiographic images can be accepted, which accounts for ≥95 % probability of microextension. For a score of ≥2, an expansion distance of 6.1 mm on radiographic images must be selected. A GTV-to-CTV expansion of 8.5 mm in radiographic images of LMCRC is required to encompass the gross tumor and all subclinical microscopic disease with 100 % accuracy.

## Discussion

The liver is the target of many metastatic cancers, particularly those of colorectal cancer. Major advances in the diagnosis and treatment of metastatic liver cancers have occurred during the last two decades. Surgery is still considered the gold-standard therapy for patients diagnosed with LMCRC because it confers the best chance of long-term survival. However, only 25 % of patients with liver metastases are candidates for curative resection [2]. The majority are not eligible for surgery due to extensive disease, multiplicity of tumor, concomitant major systemic disease, or poor hepatic reserve. During the past 20 years, SBRT has evolved as another local treatment option for primary and metastatic liver tumors. Local control rates have been improved by dose escalation protocols, whereas acceptable levels of toxicity have been maintained [6, 7, 12, 13].

To further optimize SBRT treatment, the definition of the target volume needs to be improved. Determining the degree of GTV expansion to include the CTV poses a considerable challenge to radiologists. To precisely define the limits of the target volume, the correlation between macroscopic tumor dimensions that are visible in radiological images and those that are visible in pathology specimens should be evaluated. To our knowledge, reports in the literature on this topic are scarce [14, 15]. The current study was designed as a prospective pilot study to establish all the necessary procedures to obtain a good clinicopathologic correlation between the methodologies and reproducible microextension measurements.

We found that expansions of 5.0 or 6.7 mm in microscopy specimens were required to encompass the gross tumor and any microscopic disease with 95 % or 99 % accuracy, respectively. A more important issue was to measure the tissue contraction coefficient that occurs after formalin fixation of specimens and syneresis in the automatic tissue hydroextractor. Our analysis of 66 liver carcinoma pathology specimens on slides revealed that the prepared specimen sizes were 91.4 % of the original specimens. The radiograph-slide contraction coefficient was 82.9 % (0.92 × 0.90). These calculations indicate that an expansion of at least 6.1 mm on radiographic images should be considered during radiological treatment of LMCRC.

The microextension frequency observed in the present study is in agreement with other published reports. Prospective and retrospective surgical reports describe the presence of microextensions with a large variation (2–58 %) in occurrence [16, 17]. Our results were in agreement with those of Gandhi *et al.*, who reported measurable microscopic disease (mean, 1.25 mm) in 7 of 24 (29.2 %) tumors analyzed [18]. However, the depths of microextension infiltration (0.15–38.00 mm) described in the literature are not consistent with our results, which ranged from 1–7 mm. One possible reason for the discrepancy could be that the criteria for patient selection differed in the two studies. Furthermore, it is impossible to entirely exclude whether some of our tumor nests (observed in a two-dimensional microscopic field) were not in reality attached to the primary tumor at another level, because some of the colorectal metastases had very irregular borders. We tried to correct for this weakness as much as possible by thoroughly inspecting the slices located just above and beneath the one in which we observed the microextension. In general, all the metastases included in the present study showed rather irregular shapes, and this made it difficult to establish a relationship between the irregular tumor shape and more frequent or deeper microextensions.

We found that microextension was related to the original primary tumor site, CEA levels, and multiple lesions. Reports on prognosis in colon and rectal cancers are limited. In our study, the median microextension levels in liver were significantly different depending on whether the origin of metastasis was from the colon or rectum. A possible reason for this may be the differences in their anatomical locations, which could account for different pathways in lymph node metastases and specific biological behaviors. However, further study is needed to understand the phenomenon.

There are some reports that serum CEA level is an independent risk factor for LMCRC. Patients with elevated CEA levels are more likely to develop recurrent disease and to have poorer overall survival than patients with normal CEA levels [19, 20]. Our results are in agreement with this assessment. The presence of multiple lesions is another factor that correlates with microextension, although the *P* value was only 0.045. The presence of multiple lesions suggests that the cancer cells are more highly prone to invasion.

It is unclear whether pretreatment with chemotherapy might influence metastatic microextensions. A limitation in our study is that the impact of tumor regression may not be the same after treatment with 5-fluorouracil (5-FU) or tritherapy with or without targeted agents such as the anti-VEGF monoclonal antibody bevacizumab. In our study, analysis of patients with or without chemotherapy did not identify any significant differences in microextension (*P* = 0.906). We used a specimen-slide contraction coefficient that was derived only from primary liver carcinoma specimens. We do not currently know if the LMCRC specimens would have the same coefficient. Additional research is needed to answer this question. Only 49 patients had a preoperative MRI scan in our study, which may lead to biases in the results. Most of the 129 patients included in the study had a preoperative ultrasonic sound examination. Only patients with an obscure mass or a small tumor were referred for additional examination using CT or MRI. This referral was dependent on the surgeon’s decision.

Analysis of the cumulative distribution of microinvasion in LMCRT patients with different tumor characteristics (Fig. 3) revealed that the CEA level was the best predictor of microextension. However, the pathologic stage cannot be accurately determined without surgery. Therefore, we believe that the scoring system developed in this paper, which is based on factors that can be accurately determined preoperatively (i.e., CEA level, original primary tumor site, and number of liver metastases), will hold greater promise for prognosis.

The average tumor size in this patient cohort was 2.99 cm. Considering that a tumor is approximated as a sphere, a reduction in the GTV-to-CTV expansion from 6.1 mm (score ≥ 2) to 2.41 mm (score ≤ 1) would reduce the CTV diameter from 4.21 to 3.47 cm. Accordingly, the CTV volumes would be reduced from 39.05 to 21.87 cm^3^ (i.e., a 50 % reduction). This suggests that the delineation of CTV could vary depending on differences in patient-specific clinical characteristics. Another important point is that the average tumor size in our cohort was very small (2.99 cm). Further studies based on our analyses will facilitate potential clinical applications of this promising methodology for conformal radiotherapy of LMCRT patients.

## Conclusions

This study showed that an expansion of 2.41 mm on radiographic images with a score of ≤1 can be accepted; however, to account for 98 % probability of microextension, an expansion of 6.1 mm on radiographic images with a score of ≥2 must be selected. A GTV-to-CTV expansion of 8.5 mm in radiographs of LMCRT patients is required to encompass the gross tumor and all microscopic disease with 100 % accuracy. Microextension distance is correlated with original primary tumor site, serum CEA levels, and multiple metastatic lesions in liver. Our multifactor scoring system, which takes CEA level into consideration, was a good predictor of microextension in our dataset.

## Abbreviations

LMCRC: Liver metastases from colorectal cancer
GTV: Gross tumor volume
CTV: Clinical tumor volume
CEA: Carcinoembryonic antigen
CA199: Carbohydrate antigen 199
SBRT: Stereotactic body radiation therapy
PBS: Phosphate-buffered saline
MRI: Magnetic resonance imaging
CT: Computed tomography
5-FU: 5-fluorouracil
